# Process knowledge graph modeling techniques and application methods for ship heterogeneous models

**DOI:** 10.1038/s41598-022-06940-y

**Published:** 2022-02-21

**Authors:** Jianwei Dong, Xuwen Jing, Xiang Lu, Jinfeng Liu, Haipeng Li, Xuwu Cao, Chenxiao Du, Jun Li, Lei Li

**Affiliations:** 1 School of Mechanical Engineering, Jiangsu University of Science and Technology, Zhenjiang, 212100 People’s Republic of China; 2 China Merchants Cruise Shipbuilding Co., LTD., Nantong, 226000 People’s Republic of China

**Keywords:** Mechanical engineering, Computer science

## Abstract

In the process design and reuse of marine component products, there are a lot of heterogeneous models, causing the problem that the process knowledge and process design experience contained in them are difficult to express and reuse. Therefore, a process knowledge representation model for ship heterogeneous model is proposed in this paper. Firstly, the multi-element process knowledge graph is constructed, and the heterogeneous ship model is described in a unified way. Then, the multi-strategy ontology mapping method is applied, and the semantic expression between the process knowledge graph and the entity model is realized. Finally, by obtaining implicit semantics based on case-based reasoning and checking the similarity of the matching results, the case knowledge reuse is achieved, to achieve rapid design of the process. This method provides reliable technical support for the design of ship component assembly and welding process, greatly shortens the design cycle, and improves the working efficiency. In addition, taking the double-deck bottom segment of a ship as an example, the process knowledge map of the heterogeneous model is constructed to realize the rapid design of ship process, which shows that the method can effectively acquire the process knowledge in the design case and improve the efficiency and intelligence of knowledge reuse in the process design of the heterogeneous model of a ship.

## Introduction

In order to face the competition from world-class shipyards, large shipyards must increase the application of information technology^1^. The design process of ship assembly and welding technology is one of the key factors that determine product cost, quality and cycle. On the one hand, after years of accumulation, the relevant knowledge in the field of ship process design has been very rich. However, as far as the extensive production management mode of the shipyard is concerned, the engineering knowledge lacks effective summary and sorting, and a large amount of knowledge is scattered in the local storage medium or in the minds of domain experts, reusability and sharing are poor, so this also reflects how to effectively manage and reuse this knowledge to achieve rapid design of assembly and welding processes has become a problem to be solved^2^. On the other hand, in the process of ship design and construction, a large number of heterogeneous models containing assembly and welding processes will be generated. If these heterogeneous models cannot be effectively expressed and managed, then the heterogeneous models containing process design information will be regarded as implicit Knowledge is stored in the database, it is difficult to be reused, and it is a huge waste for enterprises. Therefore, it is necessary to study how to construct a unified expression model for heterogeneous models.

In order to overcome the above problems, the process knowledge graph modeling method oriented to heterogeneous models is proposed. First, the process knowledge graph is constructed by using heterogeneous model information and assembly welding process knowledge. Then, the preliminary unified semantic representation is obtained, through the multi-strategy similarity ontology mapping method guided by the knowledge graph. Finally, invisible semantics are obtained by case-based reasoning, and the similarity of matching results is checked.

The rest of this article is arranged as follows: In “Literature review” section provides related work. In “Overviews of the proposed method” section, the basic concepts of the design of this paper are introduced, and the proposed methods are described. In “Knowledge model construction based on process knowledge graph” section, a knowledge model based on the process knowledge graph and a case-based reasoning system is constructed respectively. In “Case study and discussion” section, the effectiveness of the method is verified according to the selected model. Finally, we discuss directions for future research and present conclusions about the proposed method in “Conclusion and future works” section.

## Literature review

Along with the continuous development of CAD system in the field of ship construction and the widespread application, a large number of heterogeneous model and the associated process data and knowledge continuously generated and stored in the knowledge base of the enterprise, and for the unity of the heterogeneous model representation method is on the increase, our work inspired by recent progress in several different areas^3^, such as the knowledge map and knowledge reuse research, we will review below.

### Knowledge graph

The concept of knowledge graph was formally proposed by Google in May 2012^4^. Its original purpose was to improve the capabilities of search engines, improve the quality of search results and enhance the user’s search experience. In essence, knowledge graph is a way of revealing the relationship between entities. The semantic network can continue to formally describe things in the real world and their relationships. After 2013, with the continuous development of intelligent information services and applications and the effective management of heterogeneous and dynamic data, knowledge graphs have received widespread attention in specific fields such as academia and industry.

In academia, Hu^5^ and others studied the application of knowledge graphs in information science and introduced the necessity, current situation, development trend, and knowledge graph receipt tools and method processes of information science knowledge graph research. Hu Fanghuai^6^ studied the construction method of the Chinese knowledge graph based on multiple data sources; Giovanni Adorni and Frosina Koceva^7^ constructed knowledge representation tools for educational theory based on subject structure and representation method. Jacopo Urbani and Ceriel Jacobs^8^ studied the method of enriching query results by top-down rule-based reasoning on large RDF knowledge bases.

In other specific fields, research on knowledge graphs has also appeared. Jintao Liu^9^ proposed a new method based on knowledge graphs to explore railway operation accidents, aiming to reveal the potential rules of accidents by describing accidents and hazards in heterogeneous networks. Maya^10^ et al. extract medical concepts from a large number of patient records, and use the maximum likelihood estimation of three probability models to automatically construct a knowledge graph. Said Fathalla^11^ described how investigations in the research field are represented semantically, thus forming a knowledge graph that describes various research questions, methods, and evaluations in a structured and comparable way. Some companies have used knowledge graphs to improve existing products and have developed prominent examples of large-scale knowledge graphs, including DBpedia^12^, Google knowledge graph^13^, Microsoft’s Satori^14^, Freebase, YAGO, and Wikidata^15^. Jayaram and Khan et al.^16^ established a "Graph Query by Example” system, automatically finds the weighted hidden maximum query graph based on the input query tuple to obtain the user's query intent. Then, the top approximate matching answer graph and answer tuple can be effectively found and arranged.

However, the above knowledge graphs are mainly concentrated in the public domain, and the research on the field of ship construction is relatively limited. There is still no unified expression form for heterogeneous models based on knowledge graphs, but it is foreseeable that this technology will be used in the field of ship construction^17^. The knowledge representation and application aspects of the company have great potential. We will use the existing research results to realize the construction of process knowledge graph models for heterogeneous models.

### Domain ontology and case-based reasoning

Ontology^18^ is derived from the ontology of philosophy and is used to describe the objective existence in the world. It is a systematic explanation or explanation of objective existence. Ontology can formalize the semantic expression of concepts, which is a common method of knowledge base construction. Blythe^19^ uses the idea of ontology to research the knowledge association of the knowledge owned by the enterprise. This research can help the enterprise realize the acquisition of domain knowledge more quickly. Maedche et al.^20^ proposed the use of ontology technology to develop an integrated enterprise knowledge management framework. Wang^21^ proposed a knowledge management (KM) framework based on the Web ontology language DAML + OIL, which makes knowledge sharing more flexible and efficient. Li Zhimin^22^ proposed a simpler ontology development and expansion and a two-stage mapping method to integrate virtual enterprise ontology. These methods can improve the comprehensiveness, scalability, reusability, and sharing requirements of the ontology knowledge organization of virtual enterprises.

Case-based reasoning (CBR) first appeared in Roger's description of dynamic memory in 1982. The core idea of CBR is to help us solve the current problems with the help of cases that have solved similar problems in the past. Its core work is to retrieve historical cases that are most similar to the current case in the case library, and then retrieve the specific information based on the current problem the solutions reached are adjusted and applied to the current situation. Chang Liyun^23^ proposed a method combining discernible matrix and mathematical logic operation to get the best attribute reduction results. Sun Yanqing et al.^24^ redefines the importance of attributes so that the algorithm can simplify attributes while maintaining expert experience. Lin and Chen^25^ proposed the use of artificial immune algorithm for attribute feature selection. After the feature is selected by artificial immune algorithm, the accuracy and effectiveness of case retrieval are improved. Shen^26^ combined grey relational analysis with a genetic algorithm to optimize the feature selection process and used the result of grey relational analysis as the initial population of genetic algorithm heuristic search. Chen Hong and Mao Hongbao^27^ used knowledge entropy to determine the feature weights of cases and verified the credibility of the method through examples. Norri^28^ conducted a comparative study on three feature weighting algorithms of genetic algorithm, rough set theory, and fuzzy inference system, and the research results proved that the fuzzy set theory is the best feature weighting method. Cunningham^29^ shows that in case-based reasoning, evaluating the similarity between cases is a key aspect of the retrieval stage.

In summary, the combination of ontology and CBR can effectively improve the performance of knowledge representation and improve the performance of the CBR system^30^. The calculation of semantic similarity is one of the hotspots of ontology research. Introducing it into CBR research can effectively improve the drawbacks of traditional CBR systems that only use attribute similarity as the main matching reference basis, thereby improving the accuracy of matching results. However, the existing research on semantic similarity has not considered the influence of concept relevance on similar results, which leads to limited knowledge matching effect. Therefore, based on existing research in the industry, this paper introduces concept relevance to improve semantic similarity to obtain comprehensive semantic similarity, to improve the accuracy and objectivity of knowledge similarity calculation; and in the ontology representation of case knowledge After that, it is clustered according to the comprehensive semantic similarity to compress the knowledge matching space and improve the matching efficiency.

## Overviews of the proposed method

In this section, we first define some basic concepts, and then briefly outline our approach.

### Basic concepts

In order to visualize the dimension of each process model in machining process, the basic concepts of process dimension, dimension completeness and standardization are condensed.

#### Definition 1

Process Element (PE). Process Element is used as a set of constraint conditions for assembly and welding process. It can be expressed as:

1 $$  PE = \{  < F > | < PE_{i}  > \} ,\quad i = 1,2,......k  $$

Among, F represents the feature in the assembly and welding process, *PE*_*i*_ represents the ith process element related to process decision-making, and *k* is the process element category, such as assembly feature type, part material, welding material, etc.

#### Definition 2

Process Knowledge (PK). Standards, rules and experience knowledge used in welding process design, such as welding equipment selection rules, process analysis rules, etc. Remember to:

2 $$  {\text{PK = }}\{ {\text{P}}_{1} {\text{,P}}_{2} ,...{\text{P}}_{i} ,...{\text{P}}_{R} \} ,\quad {\text{i = 1}},{\text{2}},...  $$

Among them, ${\text{P}}_{i}$ represents rule knowledge; ${\text{R}}$ represents the category of rule knowledge.

#### Definition 3

Process Knowledge Node Relationships (PKNR). Given the process element node *P*_*i*_ and the process knowledge node *P*_*j*_, suppose the mutual entity relationship between them is:

3 $$ \lambda_{{{\text{ij}}}} = PE(P_{i} ) \cap PK(P_{j} ) $$

If the λ_ij_ ≠ ∅, given the relationship between two nodes. In the field of assembly and welding technology, the nodes of process knowledge can be extracted by the 3D model in the process of process design. This paper constructs three types of knowledge node relations, which are hierarchy relation, reference relation, and example relation respectively. Among them, the hierarchical relationship reflects the hierarchical structure and composition relationship between the three-dimensional model feature concepts; the reference relationship reflects the concept and the concept can be matched together through the reference rule; the exemplified relationship is the instantiation of the class concept.

#### Definition 4

Process Knowledge Graph (PKG). Process knowledge graph is the entity expansion and enrichment of various process knowledge under the concept nodes and relationships, and it is a structured semantic knowledge base. Process knowledge graph *K* defines a set of *(O, I)*, where *O* stands for ontology and *I* stands for instance. Both *O* and *I* triple with the form *(s, p, o)*, *s* and *o* belong to concepts or entities, and *p* is an attribute.

### Overview of the proposed method

**Figure 1:**
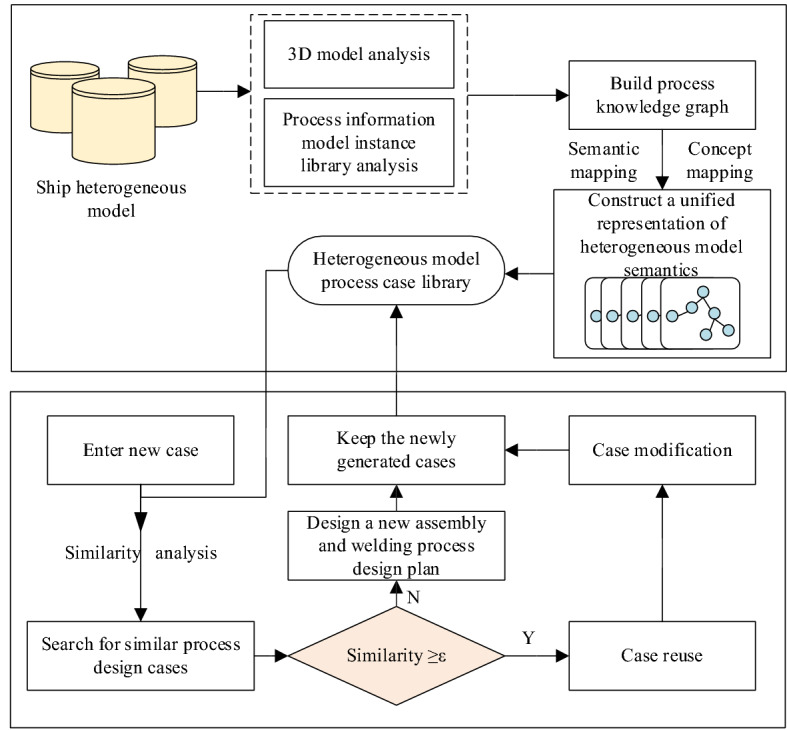
A general flow chart of the method presented in this paper.

Figure 1 shows the overall flow of the proposed method, which is guided by the process knowledge graph, the unified structured representation of heterogeneous models is realized from the perspective of process semantics. The framework consists of two parts: the process knowledge graph for building heterogeneous model of ships and the knowledge reuse system based on case-based reasoning (CBR).

Based on the analysis of the heterogeneous three-dimensional model and the process information model instance database, the process knowledge graph is constructed, and the concept mapping, semantic mapping, and other multi-strategy mapping methods are used to realize the unified structured semantic representation of the heterogeneous model.

The purpose of a case-based reasoning system is to reuse the knowledge stored in the case base with the help of a heterogeneous model process case base so that new process planning can be generated quickly. Usually, after a new case is entered, the system is a cyclic process that includes four steps: retrieval, reuse, modification, and retention.

### Ethical approval

The authors state that this paper is an original work, it has not been published in any journals, and this research does not involve any ethical issues of humans or animals.

### Consent to participate

The authors declare that this research involves no human participants and/or animals.

### Consent for publish

This work described has not been published before and has not been under consideration for publication anywhere else. Authors are responsible for the correctness of the statements provided in the manuscript and consent to be published.

### Informed consent

Consent was obtained from all individual participants included in the work.

## Knowledge model construction based on process knowledge graph

### Construct process knowledge graph of heterogeneous model

Process knowledge graph is the entity expansion and enrichment of various process knowledge under conceptual nodes and relationships and is a structured semantic knowledge network. In the field of ship process design, in order to solve the existence of heterogeneous models with conceptual mismatches and semantic inconsistencies, it is necessary to build a general process knowledge graph model to achieve a unified structured representation of ship heterogeneous models.

**Figure 2:**
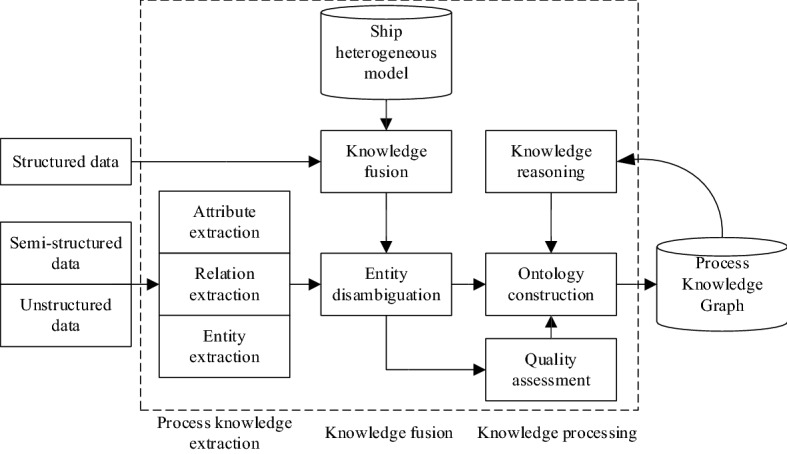
Process knowledge graph architecture.

The process knowledge graph is divided into two levels: data layer and model layer. The model layer is usually built on the data layer and stores abstract knowledge, rules, and patterns. The actual data is stored by the data layer. The overall architecture of building process knowledge graph for heterogeneous ship model is shown in Fig. 2. The part in the dashed box in the figure is the process of constructing the process knowledge graph and also the process of updating the process knowledge graph. The construction process of knowledge graph is based on the acquired heterogeneous model of ships, and a series of automatic or semi-automatic technical means are adopted. The knowledge elements are extracted from the heterogeneous model and then the knowledge is processed. After knowledge fusion and knowledge processing, it is stored in the process knowledge graph. Finally, the model layer and the data layer are linked through services to form a large process knowledge graph. The construction of the process knowledge graph is an iterative construction process. As can be seen from the figure, each round of iteration includes the following three stages.

#### Assembly and welding process knowledge extraction

**Figure 3:**
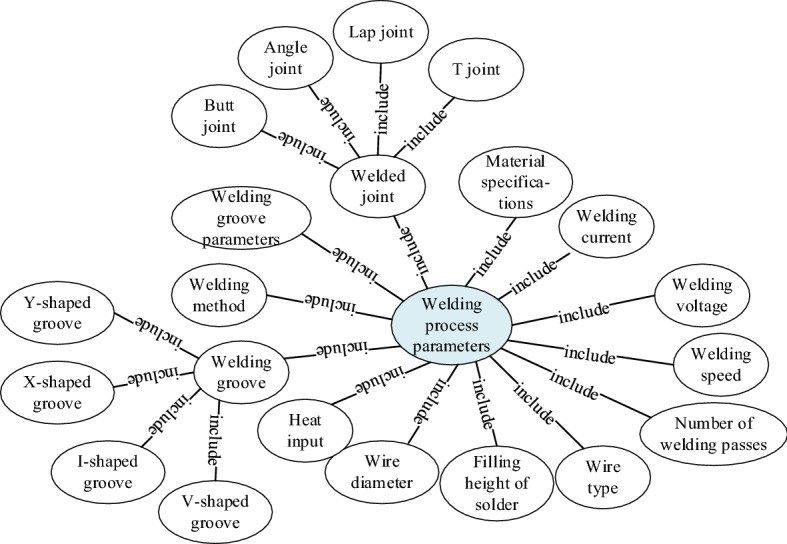
Entity extraction of welding process parameters in heterogeneous model.

**Figure 4:**
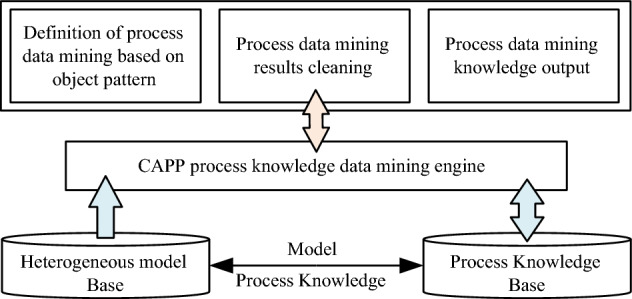
Process data mining method based on object mode.

The extraction of assembly and welding process information is the first step in the construction of process knowledge graph. The key issue is to extract entities, attributes and relationships from heterogeneous models of ships with different structures, and convert them into structured data. The process knowledge extraction in this paper includes attribute extraction, relation extraction, and entity extraction. Entity extraction refers to automatically identifying named entities from heterogeneous model data. The quality of entity extraction has a great influence on the efficiency and quality of subsequent knowledge acquisition, so it is the most basic and key part of the knowledge extraction of the assembly and welding process. As shown in Fig. 3, take the welding process parameters as an example. In order to link the extracted knowledge to the process knowledge graph, it is also necessary to extract the association relationships between entities and connect the entities through the relationships. Relation extraction is to solve the basic problem of how to extract the relations between entities. The purpose of attribute extraction is to collect attribute information of specific entities from different heterogeneous model information. The method of data mining is used to directly mine the relationship pattern between entity attributes and attribute values from heterogeneous model information to realize the positioning in the model. The object-oriented method is used to establish the process knowledge data model of the heterogeneous models in the CAPP system, and the knowledge base, knowledge logic and the process database driven by the object model are combined to realize the analysis and acquisition of process knowledge. The foundation of its application lies in the establishment, application and maintenance of the process knowledge base. This data mining method has low requirements for objects and is suitable for heterogeneous models in ship construction. As shown in Fig. 4, it is a data mining method based on process knowledge base and heterogeneous models.

#### Knowledge fusion and knowledge processing

Through the knowledge extraction of the assembly and welding process, the goal of obtaining entity, relationship, and entity attribute information from unstructured and semi-structured data is realized. However, this knowledge can contain a lot of redundancy and error information, and the relationships between data are flat and lack logic and hierarchy. Therefore, it is necessary to clean and integrate it to form structured data in the knowledge base.

Knowledge fusion includes two parts, namely entity link, and knowledge fusion. Through knowledge fusion, ambiguity and misconceptions can be eliminated, thereby ensuring the quality of knowledge. Entity linking refers to the operation of linking the entity object extracted from the text to the corresponding correct entity object in the knowledge base. The process of entity linking is: extracting entity referents from model information through entities; Whether the entity with the same name in the knowledge base and it represent different meanings is judged, and whether there are other named entities in the knowledge base and it represents the same meanings; After confirming the correct entity object in the knowledge base, the entity reference necklace is connected to the corresponding entity in the knowledge base.

Through the knowledge extraction of the assembly and welding process, knowledge factors such as entities, relationships and attributes can be extracted from heterogeneous models. After knowledge fusion, the ambiguity between entity referents and entity objects can be eliminated, and basic fact expressions can be obtained. However, the fact itself is not equivalent to knowledge. It needs to go through the process of knowledge processing to obtain the final structured and networked knowledge system.

**Figure 5:**
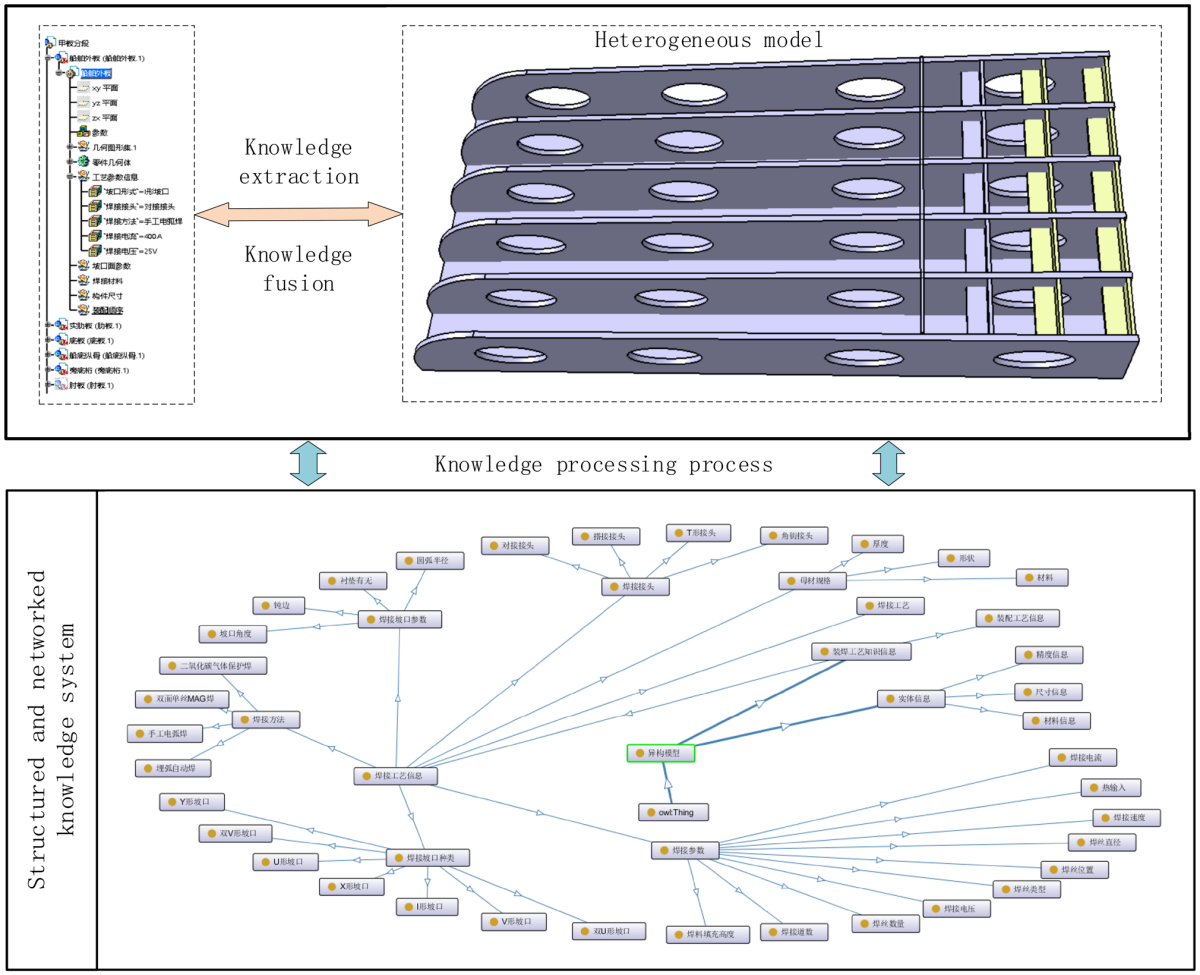
Based on the process knowledge graph to express the process knowledge contained in the heterogeneous model.

Taking the heterogeneous model in Fig. 5 as an example, the process knowledge contained in the heterogeneous model is expressed through processes such as knowledge extraction, knowledge fusion, and knowledge processing.

### Unified semantic expression of heterogeneous models

The unified semantics of the ship heterogeneous model is an expression form in which the concepts in multiple ontologies are established correspondingly, and the multiple ontologies are linked together through mapping. At the same time, the similarity of concepts is evaluated, the mapping rules are formulated according to the similarity results, and the mapping rules are corrected to ensure that the unified semantic representation of heterogeneous models is realized in the process knowledge graph.

**Figure 6:**
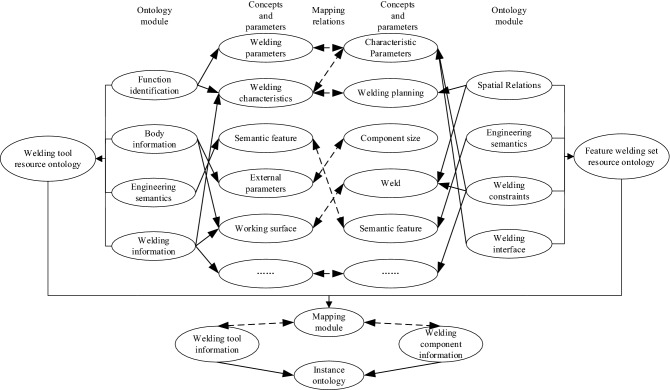
Mapping relationship between ontology.

As there are related concepts and semantic connections between the various process knowledge ontology contained in the ship heterogeneous model, the mapping relationship between the ontology is established. Taking the welding tool resource ontology and the feature welding set resource ontology as an example, the mapping *f* between the welding tool resource ontology *W* and the feature welding set resource ontology *T* is represented by the set *M* = *{m}*, where m represents the basis of a five-tuple, the mapping unit can be written in the form *e* = *(g, u, v, r, s)*. g is the unique identifier of the mapping unit; *u* and *v* are elements in *W* and *T* respectively; *r* represents the relationship between *u* and *v*; *s* is used to identify the similarity of the ontology mapping. Thus, the mapping relationship between the concepts and parameters in the welding tool resource ontology and the feature welding set resource ontology is established. The mapping relationship among connection tools, feature welding sets, and instance ontology is shown in Fig. 6. The semantic similarity of the concepts in the heterogeneous model is calculated through the corresponding mapping rules, to ensure that the unified semantic expression of the ship heterogeneous model is realized in the process knowledge graph.

### Knowledge reuse technology based on case-based reasoning

After the construction of the heterogeneous ship model case base is completed, the assembly and welding process decisions extracted previously are recorded and stored. A case-based reasoning (CBR) method is needed to retrieve and reuse assembly and welding process knowledge stored in heterogeneous ship models. In the CBR system, the existing welding process information is retrieved by calculating the similarity degree. Similarity can be divided into local similarity and global similarity. Local similarity refers to the similarity between two attributes, while global similarity refers to the similarity between two cases.

**Figure 7:**
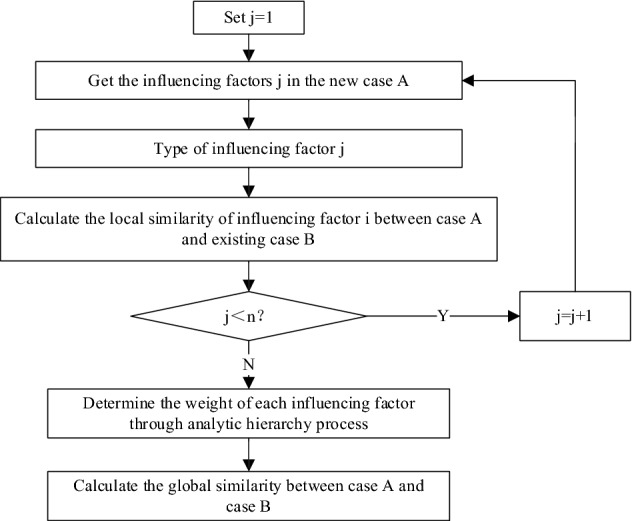
The method of calculating local similarity and global similarity.

Figure 7 shows the calculation method of local similarity and global similarity between the new case A and the existing case B, where j represents the serial number of influencing factors and n represents the total number of influencing factors. Once the influencing factors of ship heterogeneous model cases (D_1_, D_2_,…D_n_), the similarity of these attributes can be calculated by the nearest neighbor algorithm. Through the analytic hierarchy process, according to the importance of each factor to the case, each factor is assigned a weight, and then all the local similarities and their respective weights are considered, and the overall situation of the new case A and case B in the knowledge base can be calculated Similarity. In the process of using the analytic hierarchy process, a comparison matrix is created based on the input of the decision-maker, which gives the relative importance between the two influencing factors. Experts need to make judgments on factor X relative to factor Y. The more important the factor, the higher the score. The score is generally set to 1–9. If the importance of X relative to Y is set to 3, the importance of Y relative to X is 1/3. By calculating the geometric mean and standardizing it to obtain the relative weight associated with each influencing factor.

The similarity between the two cases is the global similarity, which can be defined as the weighted sum of the similarity of each influencing factor. The global similarity between the new case and the existing case can be calculated by the following formula.

4 $$ Sim(A,B) = \sum\limits_{j = 1}^{n} {\theta_{p} Sim(X_{j} ,Y_{j} )} $$

Among them, *Sim(X*_*j*_*,Y*_*j*_*)* represents the local similarity between the j-th influencing factor *X*_*j*_*,Y*_*j*_ between the new case A and the existing case B, and *Sim(A,B)* is the local similarity between the new case A and the existing case B The global similarity. *n* represents the number of influencing factors. *θ*_*p*_ represents the weight of the p-th influencing factor.

For the calculation of local similarity, there are different calculation methods for different types of influencing factors: enumerated factors, numerical factors, and irrelevant factors.

For numerical influencing factors that can be identified by numerical values, the following formula can be used to express:

5 $$ Sim(X_{j} ,Y_{j} ) = \frac{1}{{1 + |X_{j} - Y_{j} |}} $$

The attribute domain of enumerated influencing factor similarity is generally enumerated, that is, any two values of influencing factors correspond to a local similarity. Attributes such as assembly requirements, welding methods, and construction types have this type of local similarity.

6 $$ Sim(X_{j} ,Y_{j} ) = 1 - \frac{{|X_{j} - Y_{j} |}}{M} $$

The irrelevant factor means that there is no connection between the different values of the influencing factors. If the two influencing factors are the same, the local similarity is 1; otherwise, the local similarity is 0.

7 $$ Sim(X_{j} ,Y_{j} ) = \left\{ {\begin{array}{*{20}c} {1,} & {X_{j} = Y_{j} } \\ {0,} & {X_{j} \ne Y_{j} } \\ \end{array} } \right. $$

## Case study and discussion

In the construction of modern ships, heterogeneous models occupy an important proportion. The double bottom segment is a typical heterogeneous model. Through the study of double bottom segmentation, the process knowledge graph of the double bottom segment is constructed to verify the proposed feasibility of the method.

**Figure 8:**
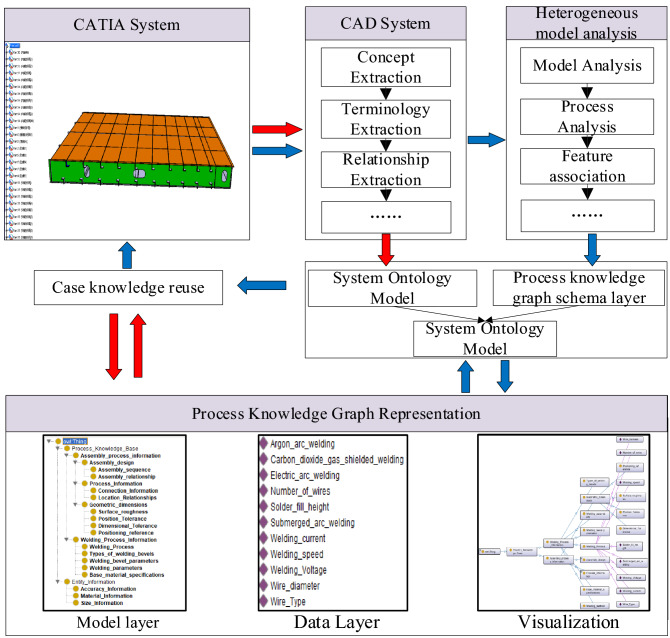
Process knowledge graph modeling system for ship heterogeneous model.

Figure 8 shows the process knowledge graph modeling prototype system for ship heterogeneous models constructed in this paper, it mainly includes two main stages: construction of heterogeneous model process knowledge graph and unified semantic generation, and case knowledge reuse. It also includes CATIA platform information extraction, heterogeneous model, and process information model analysis, which provides support for heterogeneous model information extraction and process knowledge graph construction. It can be seen from Fig. 7 that, firstly, the system ontology model is constructed by extracting the inherent concepts, terminology, and relationships of the system through the CATIA platform. Secondly, by analyzing the heterogeneous model case library and mapping related concepts, the model layer of the process knowledge graph is established. Then, the multi-strategy ontology mapping of the Protégé platform is used to generate a process knowledge graph with a unified semantic expression of heterogeneous models. At the same time, through the mutual transformation of process knowledge graph, can realize knowledge reuse and data exchange.

### Build heterogeneous model process knowledge graph

The construction of the process knowledge graph is divided into two stages. First, in order to ensure the accuracy and uniformity of concepts in the model layer of process knowledge graph, a top-down approach is adopted to map the features and process knowledge concepts to the process knowledge graph model layer to form the basic model layer of the process knowledge graph model. Since the assembly process decision is closely related to the coupling relationship and the heterogeneous model contains the macro process. Therefore, to ensure the integrity of the process knowledge graph, the relationship attributes resolved by the heterogeneous model are added to the basic model layer of the process knowledge graph to form an extended model layer of the process knowledge graph. Secondly, with the increase of process information model instance data, the process knowledge graph can be updated and expanded by extracting process entity concepts and process entity relationships.

Taking the heterogeneous model of the ship's double bottom segment as shown in Fig. 8 as an example, the process knowledge graph is constructed through the following steps:

- Step 1. Identify N assembly welding process characteristics Pi from the heterogeneous model.
- Step 2. In the process file of the heterogeneous model, M assembling and welding process information are traversed.
- Step 3. Through semantic analysis and lexical analysis, traverse M assembly and welding process information, and extract welding method information, welding equipment information, and assembly and welding strategy information. In this step, the relationship between the geometric features and the welding equipment involved in the current assembly and welding process will be obtained.

**Figure 9:**
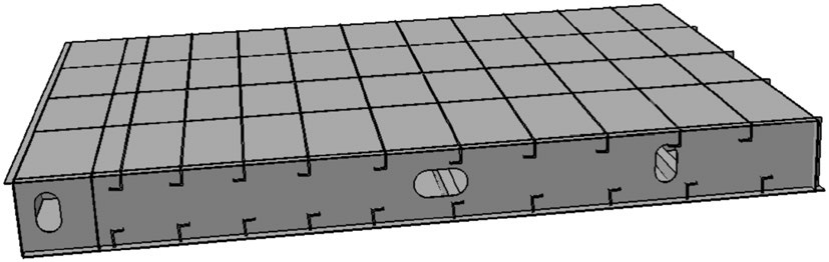
Double bottom section.

**Figure 10:**
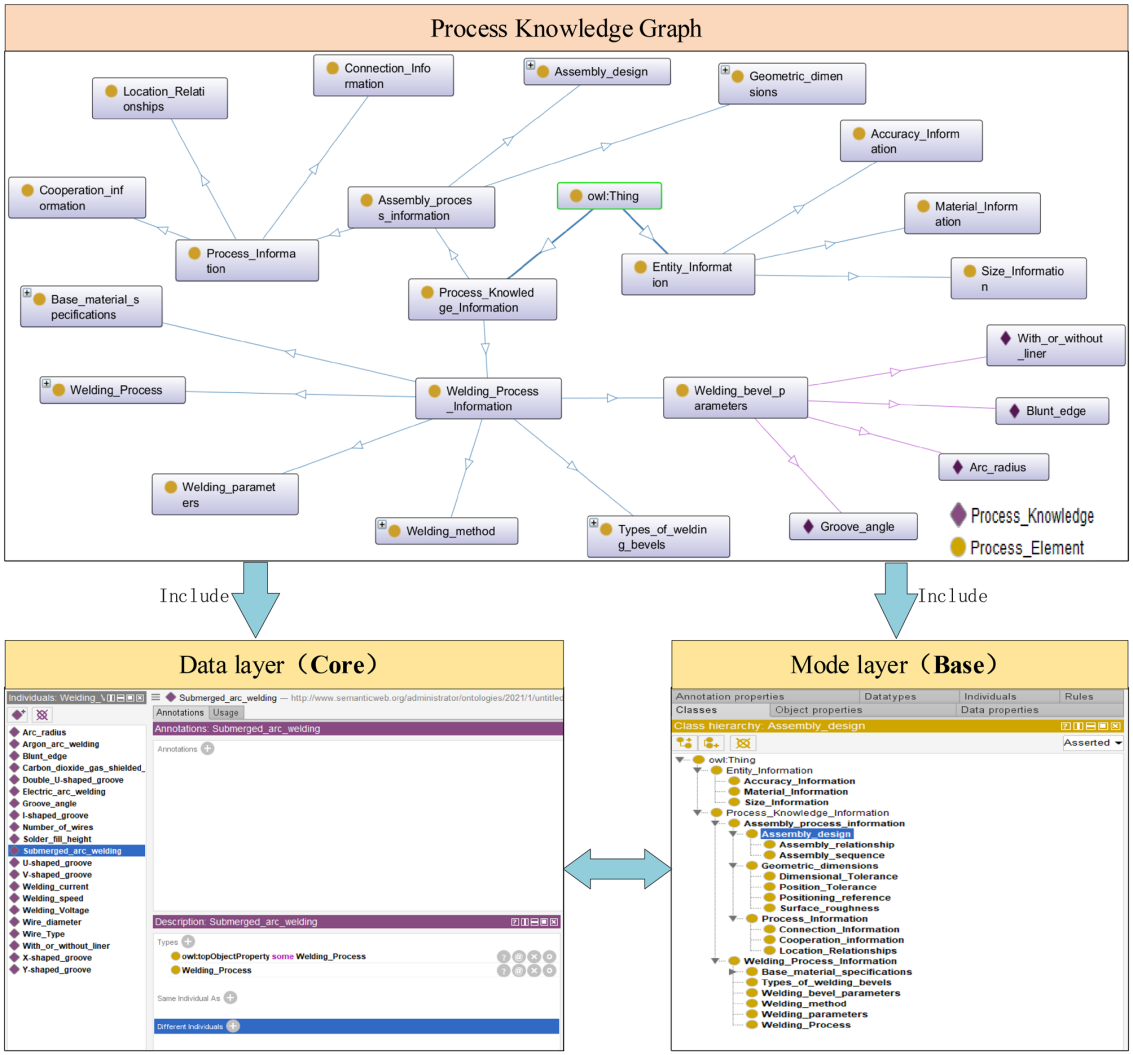
Constructing a heterogeneous model process knowledge graph.

Taking the heterogeneous model shown in Fig. 9 as an example, the process knowledge graph constructed by the above method is shown in Fig. 10. In the process knowledge map model layer, the process elements (PE) of the double bottom segment are included, such as welding material, welding speed, welding position, etc. In the data layer, there is process knowledge (PK) such as the selection of welding equipment and the type of welding groove. The Process Knowledge Graph (PKG) is composed of process knowledge nodes relationships (PKNR) composed of various process elements and process knowledge.

### Case knowledge reuse

**Figure 11:**
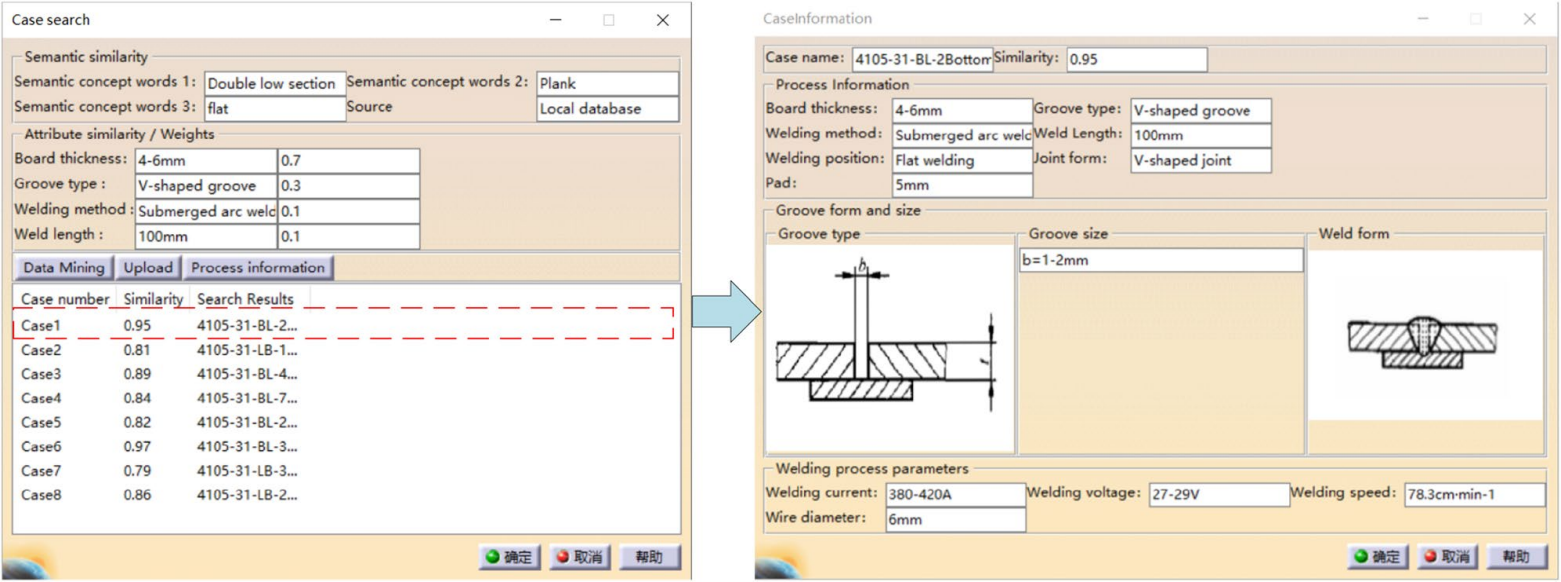
Case search and reuse module interface.

Figure 11 shows the case retrieval interface. The designer extracts the semantic words according to the retrieval intention and enters them into the semantic retrieval column, and enters the attribute value of the case knowledge in the attribute similarity check layer. The semantic concept words in this example are "plate thickness", "groove type", "welding method" and "weld seam length".

In hierarchical retrieval, the semantic similarity optimization algorithm is first used to calculate the semantic relatedness, and the semantic relatedness between domain ontologies is used to retrieve a subset of cases mapped by concept nodes, and then the numerical similarity is calculated by the attribute similarity algorithm retrieval to improve the retrieval accuracy of case knowledge. In the process acquisition interface, the system gives a sorted list of the most relevant cases, and the case structure and corresponding process design principles can be viewed through the case process information interface to realize the reuse of case knowledge.

### Discussion

Table 1 shows the comparison between the method in this paper and other related methods. It can be seen that the method in this paper and the method in^31^ both use structural elements of different granularities (for example, parts, features,); Both of them are oriented towards the manufacturing domain and have multi-level detail characteristics. The approach in^31^ is to perform a geometric decomposition of complex CAD models, aggregating technical data and topological relationships into geometric features, by proposing a machining process model for expressing the connection between machining features and machining strategies. The approach in the literature^31^ structured the representation of multi-detail level process knowledge in terms of manufacturing feature carriers, but did not consider heterogeneous models. The literature^32^ proposes the use of a metamodel as a basis for modeling manufacturing process information, which defines the information and relationships between the process of manufacturing process planning, the resources used and the products manufactured. This method is oriented to the field of machining and does not systematically consider macro and micro process knowledge. The literature^33^ proposes an ontology-based semantic retrieval method for heterogeneous 3D models, using hierarchical feature ontologies and ontology mapping techniques to generate a unified description of heterogeneous models in the form of semantic descriptors. Although the unified structured representation of heterogeneous model features is solved by the methods in^33^, the sharing and reuse of heterogeneous models cannot be realized. After comparison, this paper derives semantic expressions between process knowledge maps and heterogeneous models by proposing a multi-strategy ontology mapping method to obtain implicit semantics and check the similarity of matching results through case-based reasoning, thus realizing the reuse of case knowledge and fast design of processes.

**Table 1:** Comparison of the method in this paper with other methods.

Comparison factor	Our approach	^31^	^32^	^33^
Degree of structure	Parts/Features	Part	Manufacturing features	Design features
Model representation	PKG	UML	UML	Ontology
Level of detail	▲	▲		
Micro process knowledge	▲		▲	
Macro process knowledge	▲			
Application field	Manufacture	Manufacture	Manufacture	Design
Data set	Heterogeneous model	Process data	Isomorphic model	Heterogeneous model

## Conclusion and future works

Aiming at the problem that the knowledge contained in the existing heterogeneous models is difficult to express the influence on process knowledge reuse and sharing, a structured modeling method that uses process knowledge graph to uniformly represent heterogeneous models is proposed in this paper. Ontology is used to organize and formalize various process-related information that exists in the heterogeneous model. It provides a structured way to express and manage the process knowledge in the model. At the same time, case-based reasoning (CBR) can process the process knowledge stored in the semantic model, so it is used to retrieve and reuse the existing process knowledge to obtain the candidate welding process information.

The paper takes the construction of the process knowledge graph model of the heterogeneous model as the starting point. First, the heterogeneous model analysis is extracted through feature recognition, manufacturing semantic. And then the extracted information is fused and processed to obtain the process knowledge ontology. The method based on multi-strategy ontology mapping is used to generate a unified semantic expression of heterogeneous models. This technology can not only express the process knowledge contained in the heterogeneous model, but also effectively improve the efficiency of knowledge reuse, which is of great significance for shortening the design cycle of the ship component welding process. Overall, the main contribution of the method is to provide a knowledge model containing implicit and explicit process knowledge with multiple levels of detail based on process knowledge mapping for describing the process design intent of heterogeneous models. The method has the following features and advantages.

1. With the help of ontology modeling, ontology mapping and semantic reasoning, a unified semantic representation is established for heterogeneous models to support knowledge sharing and reuse in process co-design. This helps to compensate for the insufficient consideration of implicit process knowledge in existing feature-based models.
2. Supports multi-mode retrieval by utilizing an improved similarity metric scheme. Can promote the effectiveness of process knowledge reuse.
3. The knowledge embedded in the heterogeneous model is expressed using process knowledge mapping, thus enabling rapid reuse of assembly and welding process knowledge. Process knowledge is not only limited to the semantic level, but the process attributes are closely linked to the manufacturing semantics.

In the future, we still need to further explore some issues to improve the practicability of our method: More semantic rules will be used to improve the accuracy of semantic description; Process design information and knowledge are highly complex, and uncertainties caused by fuzzy information in the process design process need to be considered.

## Data Availability

In this submission, all the data are transparent.
